# Experimental observation of the sequence of tibial plateau chondrocyte and matrix degeneration in spontaneous osteoarthritis in Guinea pigs

**DOI:** 10.1186/s12891-021-04281-x

**Published:** 2021-04-28

**Authors:** Xiao-jian Wang, Lei Wei, Yan Xue, Rong-shan Li

**Affiliations:** 1 Department of Orthopaedic Surgery, Shanxi Provincial People’s Hospital, Taiyuan, 030012 China; 2 Precision Medicine Center, Shanxi Provincial People’s Hospital, Taiyuan, China; 3 Warren Alpert Medical School of Brown University, Providence, USA

**Keywords:** Osteoarthritis;chondrocyte, Cartilage matrix, Guinea pig

## Abstract

**Background:**

To observe the sequence of chondrocyte degeneration and matrix degradation in the superficial surface cartilage of the tibial plateau in guinea pigs with spontaneous knee osteoarthritis (OA).

**Methods:**

Sixty guinea pigs were euthanized at the ages of 8 months (*n* = 20),10 months (*n* = 20) and 12 months (*n* = 20) respectively. The degree of degeneration of the tibial plateau cartilage was evaluated by Osteoarthritis Research Society International (OARSI) score. The levels of Aggrecan,CollagenX,MMP-13 and Caspase-3 in the chondrocytes were detected by immunohistochemistry (IHC). The serum concentration of CTX-II was measured and compared. Western blot analysis was used to detect the levels of Aggrecan,CollagenX,MMP-13 and Caspase-3 in the cartilage tissue.

**Results:**

The OARSI scores both in 8-month-old group and 10-month-old group were lower than that in the 12-month-old group. The levels of Aggrecan in articular chondrocyte were higher both in 8-month-old group and 10-month-old group than that in 12-month-old group. The level of Collagen X increased with the age of guinea pigs. And the levels of MMP-13 and caspase-3 both in 10-month-old group and 12-month-old group were higher than those in 8-month-old group. The concentration of CTX-II in serum increased significantly in 12 months old group.

**Conclusion:**

The superficial chondrocytes of the tibial plateau first appeared to be hypertrophic and then apoptotic, and the matrix was further degraded when spontaneous knee osteoarthritis occurred in guinea pigs. Changes in the physiological state of chondrocytes are the initiating factors in the pathogenesis of knee OA.

## Background

Knee osteoarthritis (OA) is a degenerative disease that seriously endangers the physical and mental health of middle-aged and elderly people [1]**.** Currently, it is believed that knee OA is caused by the imbalance of articular cartilage, cartilage matrix, and subchondral bone structure and function due to the combined action of biology and mechanics and that this imbalance is followed by degeneration [2]**.**

The most obvious and typical pathological changes in knee OA are pathological changes to articular cartilage tissues: chondrocytes become hypertrophic and then apoptotic, their number is reduced, cartilage matrix is degraded, and knee OA finally occurs. The data show that articular cartilage tissues are composed of chondrocytes and extracellular matrix secreted by chondrocytes [3]**.** The extracellular matrix is mainly composed of collagenII and aggrecan [3, 4]**. **When OA occurs, the superficial cartilage tissue is first affected, the superficial cartilage tissue is broken, and longitudinal cracks appear and extend to the deep part of the cartilage tissue, which is mainly caused by the degradation of collagenII fibers in the cartilage matrix, rendering it unable to withstand physiological stress. When OA occurs, chondrocytes degenerate and then disappear due to apoptosis, leaving empty chondrocyte lacunae in cartilage tissue. When the number of chondrocytes is reduced, the extracellular matrix secreted by the chondrocytes in the cartilage tissue cannot be replenished, and the cartilage tissue further deteriorates. Sandell suggested that obese patients were prone to knee osteoarthritis, which might be due to the high concentration of inflammatory factors in joint fluid and inflammatory factors could destroy articular cartilage tissue and lead to knee osteoarthritis [5]**.** Little et al. believed that the occurrence of knee osteoarthritis was mainly due to the change of the physiological state of articular chondrocytes, which led to the decrease of chondrocytes, so stem cell transplantation could be used to treat osteoarthritis [6]**.** It is currently believed that when OA occurs, the superficial zone of cartilage tissue is the first to be affected, and the cartilage matrix and chondrocytes undergo corresponding changes [7–9]**.** However, when OA-initiating factors appear on superficial cartilage tissue, whether the cartilage matrix or chondrocytes degenerated firstly remain unclear [2, 10, 11]**.**

The aim of our study was to observe whether cartilage matrix degenerative degradation or chondrocyte degenerative apoptosis occurs first in cartilage tissue when OA-initiation factors are present and to further elucidate the natural process of cartilage degeneration.

## Methods

### Animal handing

This study was approved by the Ethics Committee of Shanxi Medical University (approval number: SXMUE2019004). Sixty 2-month-old female specific-pathogen free (SPF) grade Dunkin Hartley (DH) albino guinea pigs (Animal Experiment Center of Shanxi Provincial People’s Hospital, China) were housed in pairs and given 2 weeks to acclimate to the housing facility. Environmental conditions included a temperature of 24 °C ± 1 °C, humidity 53% ± 15%, and a 12:12 light:dark cycle with lights on at 07:00 and off at 19:00. The guinea pigs were given access to a sterilized diet and water. The guinea pigs were divided into three groups: a 8-month-old group (20 animals), which were euthanized after 6 months of feeding, a 10-month-old group (20 animals), which were euthanized after 8 months of feeding and a 12-month-old group (20 animals), which were euthanized after 10 months of feeding. All the guinea pigs were sacrificed by using intraperitoneal anesthesia with 1% sodium pentobarbital at 300 mg/kg. No animals died during the experiment. All experiments were performed in accordance with the Public Health Service Policy and the Guide to the Care and Use of Laboratory Animals.

### Histochemical studies and immunohistochemistry (IHC)

Knee joint specimens of Guinea pig were fixed and decalcified and then embedded in paraffin. The slices were then cut into 6-μm sections and mounted on slides, and the degeneration degree of tibial plateau cartilage tissue of knee joint was observed after safranin O staining and compared between groups with Osteoarthritis Research Society International (OARSI) score [12]**.**

IHC staining was used for detection of Matrix Metalloproteinase-13 (MMP-13, 1:100,

PAA099Ra01,Cloud-Clone Corp.,USA) and Caspase-3 (1:200,PAA626Ra01,Cloud-Clone Corp.,USA). We quantitatively scored the IHC results accordingto the percentage of positive chondrocytes and the staining intensity, as described below. We rated the intensity of staining on a scale of 0 to 3: 0, negative; 1, weak; 2, moderate; and 3, strong. We assigned the following proportion scores: 0 if 0% of the chondrocytes showed positive staining, 1 if 0 to 1% of the chondrocytes were stained, 2 if 2 to 10% were stained, 3 if 11 to 30% were stained, 4 if 31 to 70% were stained, and 5 if 71 to 100% were stained. We then combined the proportion and intensity scores to obtain a total score (range: 0–8), as described previously [13]**.** The results were assessed by 2 experienced pathologists a blinded manner.

### Factors detected in serum specimens

All guinea pigs were sampled for 1 ml blood by abdominal aortic puncture, which was stored sealed at 4 °C for 8 h in EDTA-coated vials, centrifuged at 6000 r/min for 15 min, and the serum was collected in Eppendorf (EP) tube for preservation in a low temperature refrigerator at − 20 °C. Serum CTX-II levels were determined by ELISA (YB-E1185GP,Alexis,Swiss).

### Western blot

The cartilage tissue samples were lysed in RIPA lysis buffer containing PMSF, protease and phosphatase inhibitors (Keygen). A certain amount of protein was mixed with loading buffer,boiled for 10 min and subjected to SDS-PAGE followed by transfer to PVDF membranes. The blots were probed with primary antibodies, after being blockedwith 5% fat-free dry milk in TBST. The relative levels of the target protein, MMP-13 and Caspase-3,to the control β-actin expressionwere determined by western blot analysis. The bound antibodies were detected with horseradish peroxidase (HRP)-conjugated secondary antibodies and visualized using the enhanced chemiluminescence reagent. Thedata were analysed by densitometric analysis using IMAGEJ software.

### Statistical analysis

SPSS 20.0 (SPSS Inc., Chicago, IL, USA) was used for the statistical analysis. All data in this studywere expressed as the mean ± standard deviation (SD). A *P* value of less than 0.05 was considered statistically significan.

## Results

### Histochemical studies and IHC

To observe and compare the degree of degeneration of the tibial plateau cartilage tissue, the OARSI scoring system was used to evaluate histological sections of guinea pig knee cartilage tissue. The results showed that the safranin O staining patterns of the tibial plateau superficial surface cartilage tissue both in the 8-month-old group 10-month-old group were intact, the surface of the superficial surface cartilage was smooth and exhibited no damage or obvious longitudinal cracks, and some superficial surface chondrocytes showed hypertrophic changes in 10-month-old group. The superficial surface cartilage of the tibial plateau in the 12-month-old group was not stained in some areas, and the superficial surface cartilage was uneven, damaged and had obvious longitudinal cracks. The superficial surface chondrocytes were significantly hypertrophic, and a certain number of empty cartilage lacunae appeared in the superficial surface.

In order to observe the biological properties of the superficial chondrocytes, IHC was used to detect the expression levels of Aggrecan protein, CollagenXprotein and MMP-13 protein, the markers of physiological state of chondrocytes, and Caspase-3 protein,a marker of apoptotic chondrocytes,in superficial chondrocytes. The results showed that the chondrocytes in the whole layer of cartilage tissue, including superficial layer, expressed more Aggrecan protein and chondrocytes hardly expressed CollagenX,MMP-13 and Caspase-3 in the 8-month-old group.

At the same time, the results also showed that a certain amount of Aggrecan protein was expressed in chondrocytes from deep cartilage tissue of 10-month-old group, but less in superficial chondrocytes. However, the levels of CollagenX,MMP-13 and Caspase-3 in superficial chondrocytes increased significantly in 10-month-old group.

**Fig. 1:**
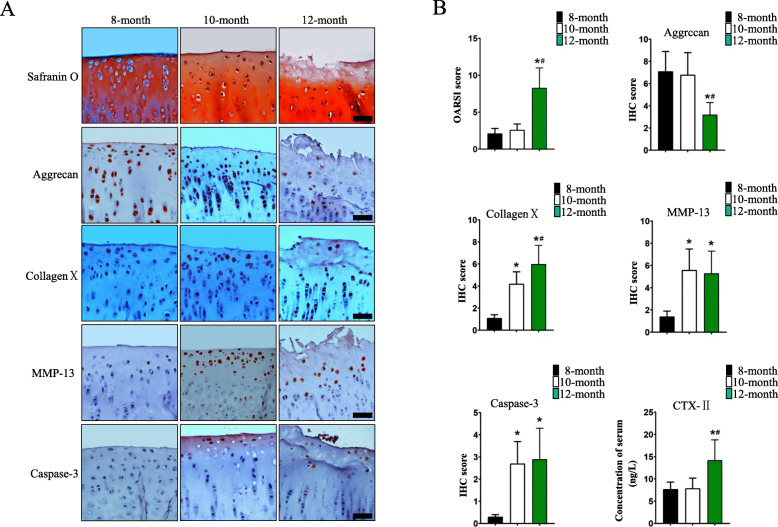
Comparison of sequences of chondrocyte and extracellular matrix degeneration in cartilage tissue. **a, b** Comparisons of safranin O staining patterns of knee tibial plateau cartilage tissue and OARSI scores, IHC results of the Aggrecan,CollagenX,MMP-13 and Caspase-3 proteins and IHC scores. Comparison of serum concentration of CTX-II. Scale = 100 μm. Statistical significance is shown for One-way ANOVA. Bars represent the mean ± SEM, *p < 0.05 compared with the 8-month-old group and ^**#**^*p* < 0.05 compared with the 10-month-old group

In addition,the superficial chondrocytes in the 12-month-old group expressed less Aggrecan protein and more CollagenX,MMP-13 and Caspase-3 in superficial chondrocytes, especially in the damaged area of the cartilage tissue. The OARSI scores of the cartilage tissues both in the 8-month-old group and 10-month-old group were lower than that in the 12-month-old group. However, there were no significant differences in the IHC scores of the MMP-13 and Caspase-3 in the superficial chondrocytes between 10-month-old group and 12-month-old group. (Fig. 1).

### Detection results of CollagenII protein degradation products in serum specimens

To detect the degree of collagen II fiber degradation in the cartilage tissue, we detected the concentration of CTX-II,which is the collagen II degradation product, in guinea pig serum. The results showed that the concentrations of CTX-II in the guinea pig serum of the 8-month-old group and 10-month-old group were significantly lower than that of the 12-month-old group (Fig. 1b).

### Western blot

**Fig. 2:**
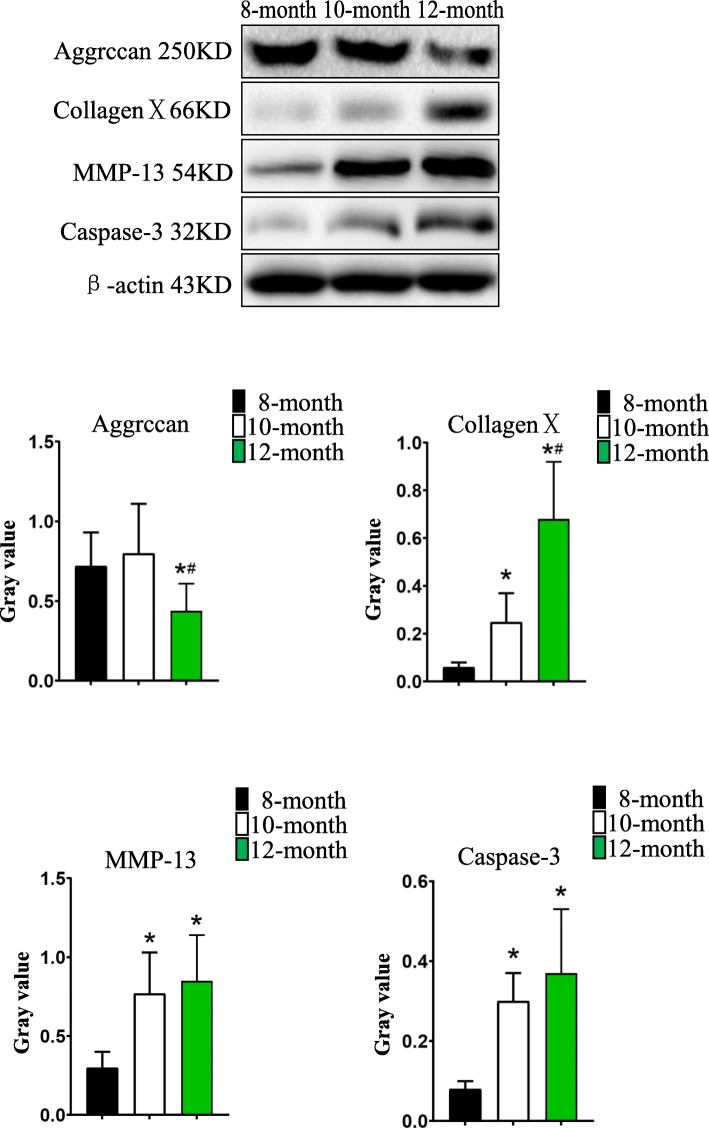
Western blot results of Aggrecan,CollagenX,MMP-13 and Caspase-3 and comparison of gray values. Bars represent the mean ± SEM, *p < 0.05 compared with the 8-month-old group and ^**#**^*p* < 0.05 compared with the 10-month-old group

**Fig. 3:**
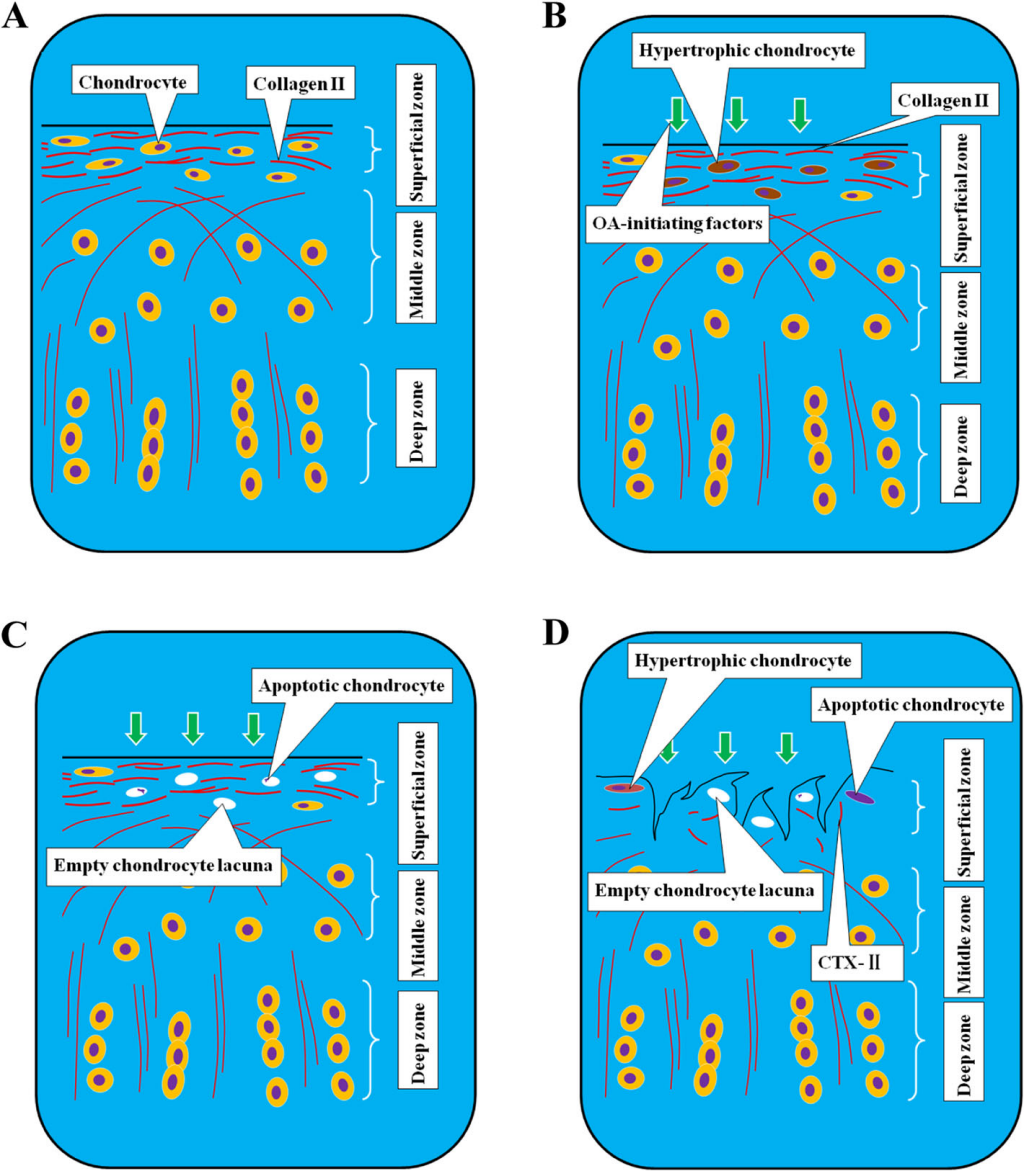
Proposed model for the sequence of chondrocyte and extracellular matrix degeneration. **a** Normal chondrocytes and normal extracellular matrix. **b** Degenerated chondrocytes, hypertrophic changes, and normal extracellular matrix when the OA-initiation factors appear. **c** Apoptosis of chondrocytes in cartilage tissue occurs,leaving empty chondrocyte lacuna. Normal extracellular matrix in cartilage tissue. **d** Chondrocyte degeneration and extracellular matrix degradation

To detect the expression levels of Aggrecan,CollagenX,MMP-13 and Caspase-3 in the tibial plateau cartilage tissue and to conduct semiquantitative analysis, we scraped the tibial plateau cartilage tissue of the guinea pigs and used western blot echnology to detect its expression levels. Articular chondrocytes in 12-month-old group expressed less Aggrecan protein and more CollagenX protein than those both in 8-month-old group and 10-month-old group. Meanwhile, the expression levels of CollagenX protein in 10-month-old group was more than that in 8-month-old group. The expression levels of MMP-13,Caspase-3 both in 10-month-old group and 12-month-old group were more than that in 8-month-old group. But there were no significant differences in the expression levels of MMP-13 and Caspase-3 between 10-month-old group and 12-month-old group (Fig. 2, Fig. 3).

## Discussion

We found that when guinea pig spontaneous knee osteoarthritis occurs,the superficial chondrocytes in the tibial plateau degenerate first, and the chondrocytes undergo hypertrophic changes, which lead to the synthesis and secretion of MMP-13 proteins. Then,the chondrocytes undergo apoptosis. Eventually, empty chondrocyte lacunae remain in the superficial cartilage tissue. Then,the number of chondrocytes in the superficial zone decreases, resulting in their inability to continue to synthesize and secrete extracellular matrix. Due to stimulation by OA-initiation factors, such as mechanical factors, collagen II fibers in the superficial zone degrade. The superficial cartilage tissue is damaged, and longitudinal cracks appear on the surface.

Knee osteoarthritis is a common degenerative disease in middle-aged and elderly people. When OA occurs, the cartilage tissue of the knee joint degenerates, chondrocyte hypertrophic changes occur, apoptosis occurs, and empty chondrocyte lacunae remain in cartilage tissue. The cartilage matrix degrades, and the cartilage tissue cannot withstand stress, resulting in injury. However, when OA-initiation factors appear, whether the chondrocytes or extracellular matrix degenerate first remains unclear [5–9]**.**Studies suggest that the extracellular matrix in the cartilage tissue degrades firstly [14, 15]**.** Cartilage tissue is similar to a concrete structure, and the collagen II fibers in the cartilage matrix are similar to supporting “steel bars” that protect the chondrocytes scattered therein. Some scholars have claimed that chondrocyte degeneration may be the initiating factor in the occurrence of OA, but there is no definite evidence to prove this idea. It is generally accepted that when OA occurs, both chondrocytes and the extracellular matrix undergo degeneration. However, when the cartilage matrix degrades, it loses its protective effect on chondrocytes, leading to chondrocyte degeneration and apoptosis. According to histology, the articular cartilage of the tibial plateau can be divided into the superficial zone, middle zone and deep zone. The superficial zone is the most superficial area of articular cartilage. The collagen II fibers in the superficial cartilage matrix are mainly transverse, and the long axis of chondrocytes is parallel to the collagen II fibers, exhibit a “flat length” shape. In addition, the superficial cartilage tissue is the first area affected when OA occurs, as has been basically concluded by academic circles. Some studies have suggested that the physiological status of superficial chondrocytes can directly or indirectly affect the status of middle zone and deep zone chondrocytes, so superficial cartilage tissues are very important in the occurrence and development of knee OA [16]**.**According to the OARSI knee cartilage degeneration score system in guinea pigs, grade 0 is normal cartilage tissue;the superficial cartilage tissue is intact, without damage, and the chondrocytes exhibit no hypertrophic changes. The grade 1 cartilage tissue is uneven and damaged, and the density of chondrocytes in the damaged area, most of which have disappeared, is extremely low. However, this system does not indicate whether chondrocytes or extracellular matrix degenerate firstly when OA changes from OARSI grade 0 to grade 1.

At present, there are many experimental methods to simulate human knee OA, such as the application of traumatic OA caused by surgery, the injection of drugs (such as papain) into the joint cavity to produce knee OA, and the use of an external fixator to brake the knee joint and cause OA. However, these methods use exogenous intervention factors, so identifying an animal model that can simulate the natural degenerative process of the human knee joint is the ideal way to study the occurrence and development of knee OA. Hartley guinea pigs are a typical animal model in OA-related studies [17]**.** Degeneration and loss of knee cartilage tissue spontaneously appear with the increase in age; in other words,spontaneous knee osteoarthritis appears. In our experiments, we found that at the age of 8 months, the cartilage tissue of knee joint was basically normal, the superficial chondrocytes did not change hypertrophy, and the cartilage matrix did not degrade. Therefore, the cartilage tissue of 8-month-old Guinea pig could be regarded as the normal knee cartilage. We also found that when guinea pigs were 10 months old, the surface of the tibial plateau cartilage tissue was intact, but the superficial chondrocytes showed significant hypertrophic changes. Some chondrocytes expressed more Caspase-3 protein, which indicated apoptosis, and there were many empty chondrocyte lacunain the superficial cartilage tissue. However, there was still no significant degradation of the collagen II fibers in the cartilage, which was confirmed by the serum concentration of CTX-II, the collagen II degradation product, in the serum. When guinea pigs were 12 months old, the tibial plateau cartilage tissue was uneven and damaged, and the superficial chondrocytes exhibited hypertrophic changes, followed by apoptosis. There was much collagen II degradation in the cartilage tissue, which was confirmed by the serum concentration of CTX-II, the collagen II degradation product, in the serum. According to the OARSI rating system, we considered the tibial plateau cartilage tissue of 12-month-old guinea pigs to be grade 1 and that of 10-month-old guinea pigs to be grade 0 ~ 1.In other words, we captured the stage when superficial chondrocytes preceded extracellular matrix degeneration during spontaneous knee osteoarthritis in guinea pigs.

Therefore, when knee OA occurs, the degeneration of chondrocytes in the superficial cartilage tissue is the initial factor. Therefore, in follow-up studies, we should focus on the changes in the physiological status of chondrocytes in cartilage tissue and consider this to be one of the key points for the early diagnosis and preventative treatment of degenerative knee OA.

## Conclusions

In conclusion,the superficial chondrocytes of the tibial plateau first appeared to be hypertrophic and then apoptotic, and the matrix was further degraded when spontaneous knee osteoarthritis occurred in guinea pigs. Changes in the physiological state of chondrocytes are the initiating factors in the pathogenesis of knee OA.

## Data Availability

The datasets used and/or analysed during the current study are available from the corresponding author on reasonable request.

## Abbreviations

OA: Osteoarthritis
OARSI: Osteoarthritis Research Society International
IHC: Immunohistochemistry
SPF: Specific-Pathogen Free
FCS: Foetal calf serum
DH: Dunkin Hartley
HRP: Horseradish peroxidase
